# Trends in Utilization of Geriatric Rehabilitation Services in Switzerland (2015-2022) – A Nationwide Cohort Study

**DOI:** 10.1093/geroni/igaf122.4222

**Published:** 2025-12-31

**Authors:** Rick Veer, Naemi Herzog, Philipp Schütz, Beat Müller, Alexander Kutz

**Affiliations:** Cantonal Hospital Aarau, Aarau, Aargau, Switzerland; University of Basel / Cantonal Hospital Aarau, Zurich, Switzerland; Cantonal Hospital Aarau, Aarau, Aargau, Switzerland; Cantonal Hospital Aarau, Aarau, Aargau, Switzerland; Cantonal Hospital Aarau, Aarau, Aargau, Switzerland

## Abstract

Population aging increases functional decline, underscoring the role of geriatric rehabilitation in restoring functional capacity after hospitalization or community referral. Yet, patient trajectories in geriatric rehabilitation remain poorly characterized. This cohort study analyzed Swiss national administrative claims (2015–2022) of adults aged ≥65 years comparing temporal trends in geriatric rehabilitation and acute-care hospitalizations. Main outcomes included causes of hospitalizations, length of stay, DRG-based cost weight, in-hospital mortality, and 30-day rehospitalization. Among 4,030,691 hospitalizations, 125,990 (3.1%) involved geriatric rehabilitation, mostly in acute-care hospitals (76.3%), followed by geriatric (20.5%) and other facilities (3.2%). Geriatric rehabilitation admissions increased by 8.3% annually (95% confidence interval [CI]: 5.2–11.5) versus 1.2% (95% CI: 0.3–2.2) in acute-care. Circulatory diseases were the leading cause of acute-care admissions (19.3%), while injuries predominated in geriatric rehabilitation (25.1%) and showed the steepest increase (+9.7% annually; 95% CI: 6.3–13.1), followed by circulatory, musculoskeletal, and mental disorders. Length of stay declined more in geriatric rehabilitation (−4.3% per year; 95% CI: −4.9-−3.7) than in acute care (−2.0%; 95% CI: −2.2-−1.9). DRG-based cost weight decreased annually by -3.09% (95% CI: −4.60%-−1.57%) but remained stable in acute care. Discharge home after geriatric rehabilitation declined by − 1.2% per year (95% CI: −1.6-−0.7), with increasing transfers to other rehabilitation and nursing homes. 30-day rehospitalization and in-hospital mortality were stable in both groups. In conclusion, the proportion of hospitalizations involving geriatric rehabilitation is rising, reflecting growing demand in an aging population. Its effectiveness for patient outcomes and health system performance warrants further evaluation.

